# Assessing Agreement between Blue-Light and Green-Light Autofluorescence of Macular Hyperautofluorescent Rings in Inherited Retinal Diseases

**DOI:** 10.1016/j.xops.2025.101026

**Published:** 2025-12-02

**Authors:** Malena Daich Varela, Nancy Aychoua, Memuna Rashid, Andre Lopes, Michel Michaelides

**Affiliations:** 1 Moorfields Eye Hospital, London, United Kingdom; 2 UCL Institute of Ophthalmology, University College London, London, United Kingdom; 3 CRUK Cancer Trials Centre, University College London, London, United Kingdom

**Keywords:** Retina, Genetics, Dystrophy, Autofluorescence, Imaging

## Abstract

**Purpose:**

To assess the agreement between blue autofluorescence (BAF) and green autofluorescence (GAF) fundus imaging in measuring the macular hyperautofluorescent (hyperAF) ring in patients with inherited retinal diseases (IRDs).

**Design:**

A prospective, within-subject agreement study.

**Subjects:**

A total of 124 patients with IRD (67% with retinitis pigmentosa, 33% with cone–rod/macular dystrophies) from Moorfields Eye Hospital were included. Mean age was 36.5 years; 60% were male. Most (99%) had a genetically confirmed diagnosis.

**Methods:**

Participants underwent BAF (Heidelberg) and GAF (Optos) imaging during the same visit. Hyperautofluorescent ring area and horizontal and vertical diameters were independently measured by 2 ophthalmologists. Agreement between imaging modalities was assessed using intraclass correlation coefficients, Pearson correlation, paired *t* tests, linear and mixed-effects regression, Bland–Altman plots, and 1-sided z-tests for equivalence within a ±10% margin.

**Main Outcome Measures:**

Comparison of hyperAF ring area and diameters between BAF and GAF.

**Results:**

Intergrader agreement was excellent (intraclass correlation coefficient: 0.93–0.98). Blue autofluorescence measured slightly larger ring areas than GAF (mean difference: 0.7 mm^2^, *P* = 0.006), while horizontal and vertical diameters were nearly equivalent (mean differences: 0.03 μm and 0.08 μm, *P* = 0.96 and *P* = 0.04, respectively). Discrepancies >10% were observed in 17% of cases in horizontal diameter, 27% of cases in vertical, and 42% of cases in area. Correlations were high (*r* = 0.98) for all metrics. Mixed-effects models including both eyes (n = 217) estimated that the area measured in GAF is 4.1% smaller than in BAF, the horizontal is approximately 0.5% smaller, and the vertical is around 2.2% smaller, with evidence of agreement within a ±6% margin at a 5% significance level.

**Conclusions:**

High correlation and consistent regression slopes were seen between BAF and GAF macular hyperAF ring. These results suggest that while the 2 modalities yield broadly comparable measurements, with BAF yielding larger values, these modalities should ideally not be used interchangeably.

**Financial Disclosures:**

Proprietary or commercial disclosure may be found in the Footnotes and Disclosures at the end of this article.

Inherited retinal diseases (IRDs) constitute a mixed group of rare disorders with a wide range of signs and symptoms. Around 1 in 2000 individuals has an IRD, and this is the most common cause of blindness in the working-age population in England.^1^^,^^2^ The 2 main types of IRD are rod–cone (also known as retinitis pigmentosa [RP]) and cone–rod dystrophies (CORD).^3^ Inherited retinal diseases have become an area of great interest within ophthalmology, genetics, retinal multimodal imaging, and artificial intelligence, with an ever-expanding number of clinical trials and novel therapeutics in the pipeline.^4^, ^5^, ^6^, ^7^, ^8^

Fundus autofluorescence imaging is a key tool to diagnose and monitor the progression of IRD, revealing the retina’s health and metabolism and providing insights into pathophysiology.^9^ Endogenous fluorophores like lipofuscin can be found in the retinal pigment epithelium (RPE), and the autofluorescence signal relates to the pace at which photoreceptor outer segments are metabolized by the RPE cells.^10^^,^^11^ A useful feature to monitor IRD is the macular hyperautofluorescent (hyperAF) ring, often seen demarcating the border outside or inside of which the disease is active.^9^

There are 3 main types of fundus autofluorescence: (1) short wavelength autofluorescence or blue autofluorescence (BAF), acquired with a 488 nm blue light that excites lipofuscin and N-retinylidene-N-retinylethanolamine (A2E), with an emission range between 560 and 700 nm; (2) near infrared (NIR), where excitation occurs at 787 nm (thereby also exciting melanin located in the RPE and choroid) and emission around 800 nm; and (3) green autofluorescence (GAF), such as used by the ultra-widefield Optos device (Optos PLC), with an excitation at approximately 514-532 nm and emission at around 540 nm.^10^^,^^12^^,^^13^ The first 2 employ a confocal scanning laser ophthalmoscope, and the third one a confocal scanning laser technology with an ellipsoid mirror.

Differences exist depending on which fundus autofluorescence is used. With all modalities the optic nerve and vessels appear dark due to lack of lipofuscin and light absorption by blood, whereas the macular appearance differs.^14^ Macular pigments such as lutein and zeaxanthin absorb short wavelength light; hence, the macula appears hypoautofluorescent in BAF, and the evaluation of the fovea may be challenging.^15^ Green autofluorescence and NIR may be better at detecting foveal changes, with the macula appearing hyperAF in NIR.^14^ These imaging modalities complement each other, as NIR appears to detect geographic atrophy and pigment migration earlier than BAF, while BAF is better at detecting areas with photoreceptor loss but intact RPE, as well as subretinal hyperreflective material, whereas GAF is an excellent tool to detect small foveal and macular changes.^16^, ^17^, ^18^ Near infrared is also useful when acquiring images through narrow pupils or opaque media.^19^

Multimodal retinal assessment in IRD is key to monitoring progression and advising on prognosis. Herein, we compare measurements of a common feature of IRDs, a macular hyperAF ring, using BAF and GAF fundus imaging and assess their agreement.

## Methods

### Study Design and Participants

This prospective, within-subject agreement study was conducted at a single ophthalmology center in the United Kingdom. Patients with IRD attending Moorfields Eye Hospital who had both BAF (55°, Spectralis, Heidelberg Engineering) and GAF (Optos) images during the same visit and had a clear and complete macular hyperAF ring were included. Patients were consecutively selected from a database of patients with IRD. Demographic characteristics such as sex at birth and age at imaging were recorded, as well as IRD phenotype and the gene involved in the diagnosis. Informed consent was obtained from all patients. Ethical approval was provided by the local ethics committee, and the study honored the tenets of the Declaration of Helsinki.

**Figure 1:**
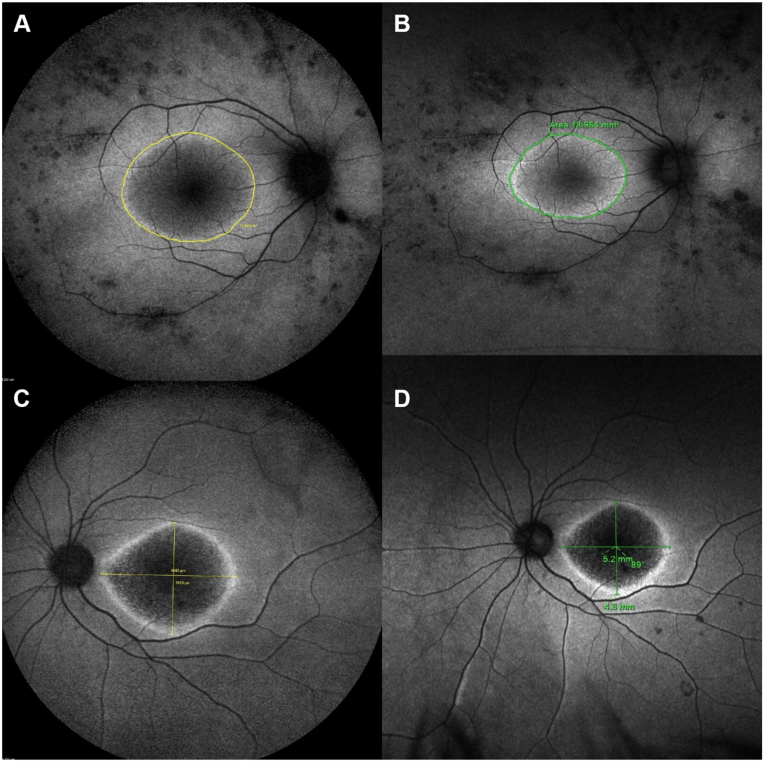
Examples of macular hyperAF ring measurements in BAF and GAF in patients with IRD. (**A** and **B**) Area of the macular ring of a patient with *USH2A*-associated RP, measured in BAF (**A**) and GAF (**B**). (**C** and **D**) Horizontal and vertical diameters of the macular ring in a patient with *GUCY2D*-associated CORD, measured in BAF (**C**) and GAF (**D**). In all cases, GAF measurements are minimally smaller than BAF. BAF = blue autofluorescence; CORD = cone-rod dystrophies; GAF = green autofluorescence; hyperAF = hyperautofluorescent; IRD = inherited retinal disease; RP = retinitis pigmentosa.

Ring area (mm^2^) and horizontal and vertical diameters (mm) were independently measured by 2 trained ophthalmologists (M.D.V. and N.A.) capturing the outer border of the area of increased signal with the imaging demarcating tools. Measurements were averaged and compared between imaging modalities (Fig 1).

### Data Analysis

We assessed intergrader agreement using the intraclass correlation coefficient. Analyses focused on right eye (OD) measurements of area, vertical diameter, and horizontal diameter by convention. Pearson correlation coefficients were used to evaluate linear associations between BAF and GAF measurements. Paired *t* tests were applied to assess mean differences between modalities, using log-transformed values to address skewness. Linear regression models were fit for each metric, with BAF regressed on GAF. Models were adjusted for age, sex, and phenotype.

To assess multiplicative effects, we also performed regressions on log-transformed data, allowing coefficients to be interpreted as percentage changes. Agreement was examined using Bland–Altman analysis for right eye measurements. We calculated absolute and percentage differences between modalities and plotted them against their averages. Limits of agreement (mean ± 1.96 standard deviation [SD]) were derived for each metric.

We also reported the proportion of measurements with percentage differences within ±10%, considered a clinically acceptable range. To account for within-subject correlation, mixed-effects models were fit using data from both eyes. Fixed effects included imaging method, age, sex, phenotype, and eye; a random intercept for patient was included. Coefficients were exponentiated to allow interpretation on a proportional scale. One-sided z-tests for equivalence assessed whether differences between methods laid within a ±10% prespecified margin. Equivalence was concluded if both 1-sided tests yielded *P* < 0.05.

## Results

### Demographics and Baseline Characteristics

One hundred twenty-four patients were included in this study; 74 (60%) were male and 50 (40%) female (Table 1). Mean age at imaging was 36.5 ± 17.2 years old (range 4–75); 18 patients were children (<18 years old), and 106 were adults. Eighty-three patients had RP (67%), and 41 had CORD or macular dystrophy (CORD/MD, 33%). One hundred twenty-three patients were genetically confirmed, and 1 was unsolved; the most common gene was *USH2A* in 25 patients, followed by *ABCA4* in 13.

**Table 1:** Patients’ Clinical Characteristics

Patients	(n = 124)
Gender (n [%])	
Female	50 (40)
Male	74 (60)
Phenotype (n [%])	
Retinitis pigmentosa	83 (67)
Cone–rod/macular dystrophy	41 (33)
Age at imaging (mean ± SD, yrs)	36.5 ± 17.2
Adults (n)	106
Pediatric (n)	18
Laterality (n)	
Right eyes	120
Left eyes	97

n = number; SD = standard deviation.

Ninety-six left eyes and 120 ODs were included. Intergrader agreement was high for all measurements, with intraclass correlation coefficients of between 0.93 and 0.98 for area, vertical, and horizontal diameters.

### Macular hyperAF Ring Metrics

The mean ± SD area of the hyperAF ring in OD with BAF was 19.2 ± 17.4 mm^2^ (median 15.5), the mean horizontal diameter was 5.0 ± 2.1 μm (median 5.1), and the mean vertical diameter was 4.1 ± 1.9 μm (median 3.9, Table 2). With GAF, the mean area of the hyperAF ring in OD was 18.5 ± 16.8 mm^2^ (median 15.4), the mean horizontal diameter was 5.0 ± 2.0 μm (median 4.9), and the mean vertical diameter was 4.0 ± 1.9 μm (median 3.8).

**Table 2:** Comparison of BAF and GAF Measurements for Macular Hyperautofluorescent Ring Area and Diameters (Right Eye)

Measure	Area (mm^2^)	Horizontal Diameter (μm)	Vertical Diameter (μm)
Descriptive statistics			
Mean (SD) BAF	19.2 (17.4)	5.0 (2.1)	4.1 (1.9)
Mean (SD) GAF	18.5 (16.8)	5.0 (2.0)	4.0 (1.9)
Mean differences (BAF – GAF)			
Raw scale, paired *t* test	0.7 (95% CI: 0.2–1.1) *P* = 0.006	0.03 (95% CI –0.02 to 0.08) *P* = 0.96	0.08 (95% CI 0.02–0.1) *P* = 0.04
Log-transformed difference	0.03 (95% CI: 0.01–0.06)	0.00 (95% CI –0.01 to 0.01)	0.02 (95% CI 0.00–0.04)
Geometric mean ratio (BAF/GAF)	1.034 (95% CI: 1.016– 1.051)	1.004 (95% CI 0.99–1.011)	1.018 (95% CI 1.005– 1.03)
Derived percentage difference	3.4% (95% CI: 1.6%– 5.1%)	0.04% (95% CI –1.0% to 1.1%)	1.8% (95% CI 0.5%– 3.0%)
Correlation and linear regression			
Pearson correlation, *P* value	*r* = 0.98, *P* < 0.0001	*r* = 0.98, *P* < 0.0001	*r* = 0.98, *P* < 0.0001
Linear regression (BAF on GAF)	BAF = 0.2 + 1.0 × GAF	BAF = 0.02 + 1.0 × GAF	BAF = 0.1 + 1.0 × GAF
Agreement			
Bland–Altman limits of agreement	–5.5 to 6.8	–0.74 to 0.80	–0.72 to 0.88
% of measurements within ±10% agreement	58%	83%	73%

BAF = blue autofluorescence; CI = confidence interval; GAF = green autofluorescence; SD = standard deviation.

The mean ± SD area of the hyperAF ring in left eye with BAF was 22.4 ± 17.8 mm^2^ (median 17.0), the mean horizontal diameter was 5.4 ± 1.9 μm (median 5.2), and the mean vertical diameter was 4.6 ± 1.8 μm (median 4.1). With GAF, the mean area of the hyperAF ring in left eye was 21.4 ± 16.9 mm^2^ (median 16.7), the mean horizontal diameter was 5.3 ± 1.8 μm (median 5.2), and the mean vertical diameter was 4.4 ± 1.8 μm (median 4.2).

Both eyes of 92 patients were included, and the mean differences ± SD between both eyes of the same patient were 0.6 ± 13.5% in area in BAF (median 0.2%) and 0.3 ± 11.8% in GAF (median 0.2%); 0.9 ± 8.3% in vertical diameter in BAF (median 1.1%) and 0.7 ± 8.3 μm in GAF (median 0.8%); and 1.5 ± 7.3% in horizontal diameter in BAF (median 0.9%) and 1.5 ± 6.5 μm in GAF (median 1.8%).

### Agreement between BAF and GAF

Table 2 summarizes the agreement between BAF and GAF measurements of macular hyperAF ring area, horizontal diameter, and vertical diameter in the right eye.

### Area

For measurements of the area, BAF values were slightly higher than GAF, with a mean difference of 0.7 mm^2^ (95% confidence interval [CI]: 0.2–1.1). The log-transformed difference was small but statistically significant (0.03, 95% CI: 0.01 to 0.06, *P* = 0.006), corresponding to a geometric mean ratio of 1.034. This suggests that BAF measurements were on average 3.4% higher than GAF (95% CI: 1.6%– 5.1%).

**Figure 2:**
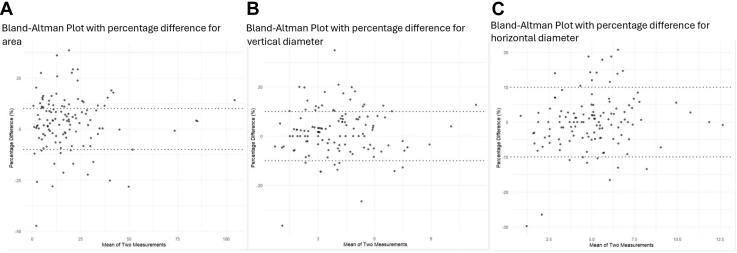
Bland–Altman plot comparing measurements between GAF and FAF in (**A**) area, (**B**) vertical diameter, and (**C**) horizontal diameter. The x-axis represents the mean of the 2 measurements, and the y-axis represents the percentage difference between them (BAF - GAF). The dashed lines represent the 10% agreement. Differences within ±10% were seen in 58% of measurements of area, 73% of measurements for vertical diameter, and 83% of horizontal diameter. Intraclass correlation coefficient for the area between was 0.983 (95% CI 0.975–0.99), for vertical diameter 0.976 (95% CI 0.97–0.98), and for horizontal 0.98 (95% CI 0.97–0.99). BAF = blue autofluorescence; CI = confidence interval; FAF = fundus autofluorescence; GAF = green autofluorescence.

The correlation between modalities was high (*r* = 0.98), and the slope of the regression line (1.0) indicated a proportional relationship with minimal systematic bias (intercept = 0.2). However, Bland–Altman analysis showed wide limits of agreement (–5.5 to 6.8 mm^2^), and 58% of measurements were within ±10% of agreement. Discrepancies >10% were observed in 42% of cases (29% where BAF was greater than GAF, 13% where GAF was greater than BAF; Table 2 and Fig 2), in particular in 29 eyes with RP (36.3% of all RP cases) and 21 with CORD (52.5%).

### Horizontal Diameter

For measurements of the horizontal diameter, the mean difference was negligible (0.03 μm, 95% CI –0.02 to 0.08, *P* = 0.96), and the log-transformed difference centered around zero. The geometric mean ratio was 1.004 (95% CI: 0.99 to 1.011), with the CI including 1, indicating no statistical evidence of a systematic difference between modalities.

**Figure 3:**
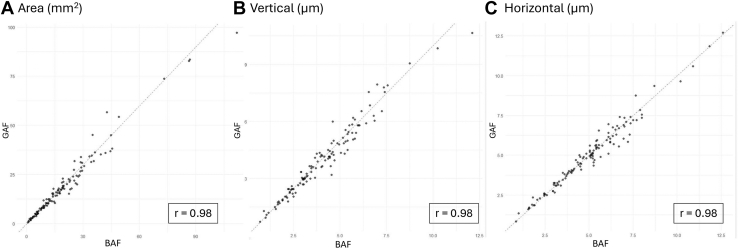
Scatter plots showing high positive correlation between BAF and GAF area (**A**), vertical diameter (**B**), and horizontal diameter (**C**). Pearson correlation coefficients indicate very strong relationships (*r* = 0.98) for all 3 variables and *P* values < 0.0001. These results demonstrate a statistically significant, near-perfect linear relationship between BAF and GAF. BAF = blue autofluorescence; GAF = green autofluorescence.

The Pearson correlation was high (*r* = 0.98, Fig 3), and linear regression showed a slope of 1.0 and intercept of 0.02, supporting a consistent relationship across the measurement range. Agreement was strong, with 83% of values within ±10% and narrow Bland–Altman limits (–0.74 to 0.80 μm), suggesting that BAF and GAF may be used interchangeably for horizontal diameter measurements. However, discrepancies >10% were observed in 17% of cases (10% where BAF was greater than GAF, 7% where GAF was greater than BAF; Table 2 and Fig 2), in 13 eyes with RP (16%) and 8 with CORD (20%).

### Vertical Diameter

For the vertical diameter, the mean difference was small and not statistically significant (0.08 μm, 95% CI 0.02–0.1, *P* = 0.04), and the geometric mean ratio was 1.018 (95% CI 1.005 to 1.030), indicating that BAF measured values were on average 1.8% higher than GAF (95% CI 0.5%–3.0%).

The correlation was high (*r* = 0.98), and the regression equation (slope = 1.0, intercept = 0.1) indicated good agreement with minimal measurement discrepancy between modalities. However, limits of agreement were slightly wider than for the horizontal diameter (–0.72 to 0.88 μm), and 73% of measurements were within ±10%, suggesting moderate agreement between BAF and GAF. Discrepancies >10% occurred in 27% of cases (19% where BAF was greater than GAF, 8% where GAF was greater than BAF; Table 2 and Fig 2), in 21 eyes with RP (26%) and 12 with CORD (30%).

### Analysis of Both Eyes

In the analysis including both eyes (n = 217) and accounting for the correlation between eyes from the same patient (random intercept), the linear mixed-effects model—adjusted for sex, age, and phenotype—estimated that the area measured in GAF is 4.1% smaller than in BAF (95% CI 2.2%–5.9%, Supplementary Table 1, available at www.ophthalmologyscience.org). The exponentiated coefficient was 0.959 (95% CI 0.940–0.978), lying within the ±10% margin (0.90–1.10). Therefore, at a 5% significance level, there is evidence of agreement between BAF and GAF derived areas, with a small but statistically significant difference.

For the diameter, the linear mixed-effects model estimated that in GAF the horizontal is approximately 0.5% smaller than in BAF (95% CI –1.6% to 0.6%), and the vertical is around 2.2% smaller than in BAF (95% CI –3.4% to –0.7%), both within a ±4% margin, at a 5% significance level. For the lower margins and the upper margin, the *P* values were <0.0001 in all measurements, so we can conclude equivalence between BAF and GAF.

### Proposed Equations

Linear regression derived equations to estimate area are BAF = 0.2 + 1.0 × GAF, horizontal diameter BAF = 0.02 + 1.0 × GAF, and vertical diameter BAF = 0.1 + 1.0 × GAF (Table 2).

## Discussion

In this prospective agreement study, we found high correlation and consistent regression slopes between BAF and GAF fundus imaging for measuring the macular hyperAF ring. These results suggest that the 2 modalities yield broadly comparable measurements, especially for horizontal diameter and vertical diameter.

Analyzing both eyes, the macular hyperAF ring was minimally smaller in GAF than in BAF in all parameters. At a 5% significance level, there is evidence of agreement between BAF and GAF within a ±6% margin, indicating that the differences, despite sometimes being statistically significant, are very small. Nevertheless, despite the statistical agreement, a substantial proportion of measurements for area fell outside the ±10% margin (42%), indicating meaningful differences at the individual level, minimally more frequent in CORD versus RP. This level of disagreement, particularly for area, should be considered when interpreting or comparing measurements across imaging modalities in clinical and research contexts.

Looking at the 3 linear regressions (area, horizontal diameter, and vertical diameter), the slopes are very close to 1, which suggests strong proportional agreement between the imaging modalities, with the small shifts in the intercept possibly indicating minor absolute differences yet general relative agreement.

Green autofluorescence has also been found to display smaller areas than BAF when measuring geographic atrophy in age-related macular degeneration.^20^^,^^21^ This difference could be due to image distortion, machine calibration, multiple image averaging in BAF, or differences in the absorption of GAF and BAF wavelengths.^20^

There is a need to be able to fully utilize real-world data of patients with rare diseases to better understand disease progression, enable long-term clinical monitoring, characterize large patient populations, act as natural history control data, and draw genotype-phenotype correlations.^22^ One limitation is the use of different imaging modalities and the lack of knowledge regarding how these can be analyzed conjunctly. In this study, the association between BAF and GAF imaging of macular hyperAF rings in patients with IRD has been explored.

Green autofluorescence has been reported to often be more comfortable to obtain than BAF, with less energy delivered.^21^ Also, being fast and easily acquired, it could be suitable for self-service retinal photography, potentially reaching a larger proportion of the community.^23^ Moreover, it allows full-field retinal imaging compared to current BAF devices. Although using the same device for disease monitoring is the gold standard, the equations proposed herein may facilitate trend visualization between modalities, maximize utility of acquired imaging data, and increase our understanding of IRD.

Of note is the finding that the mean differences in all macular ring metrics between eyes were <2% with both imaging modalities, reinforcing the key feature of symmetry in IRD.^3^

The study strengths include a large cohort of genetically proven patients with IRD and multimodal imaging done on the same visit at the same hospital by the same technician per patient. The limitations include different device operators between patients and versions of the devices. Although there are often long intervals between clinical imaging sessions, photobleaching may have occurred and affected the ring parameters, especially when BAF was done before GAF. The exploratory predictive equations require validation in an external dataset and possibly calibration to be able to reliably predict the measurements.

In conclusion, measurements of the macular hyperAF ring were minimally larger in BAF than in GAF imaging. The derived models provide a basis for translating measurements between GAF and BAF, which may facilitate their interchangeable use for monitoring disease progression.
