# Supplementary material for: Assessing Agreement between Blue-Light and Green-Light Autofluorescence of Macular Hyperautofluorescent Rings in Inherited Retinal Diseases
Source: Ophthalmol Sci. 2025 Dec 2;6(3):101026. doi: 10.1016/j.xops.2025.101026 (PMC12876574; doi:10.1016/j.xops.2025.101026)
Supplement: Supplementary Table 1 [file mmc1.pdf]

Supplementary Table 1: Results of linear mixed-effects model

	Fixed effects	Log-scale	Exponentiated			Percent change		
		Estimate	Estimate	2.5% CI	97.5% CI	Estimate	2.5% CI	97.5% CI
**Area**	(Intercept)	2,49	12,05	7,79	18,66	NA		
	Imaging (GAF)	-0,04	0,96	0,94	0,98	**-4,10%**	**-5,94%**	**-2,21%**
	Age	0,00	1,00	0,99	1,01	0,30%	-0,71%	1,32%
	Sex (M)	0,20	1,22	0,87	1,72	22,46%	-13,00%	72,20%
	Phenotype (CORD)	-0,34	0,71	0,49	1,03	-29,05%	-51,20%	2,83%
	Eye (OS)	0,01	1,01	0,99	1,03	0,74%	-1,37%	2,89%
**Horiz**	(Intercept)	1,49	4,43	3,58	5,49	NA		
	Imaging (GAF)	-0,01	0,99	0,98	1,01	**-0,51%**	**-1,58%**	**0,56%**
	Age	0,00	1,00	1,00	1,01	0,08%	-0,41%	0,57%
	Sex (M)	0,11	1,11	0,94	1,32	11,36%	-5,76%	31,59%
	Phenotype (CORD)	-0,17	0,84	0,70	1,01	-15,64%	-29,66%	1,18%
	Eye (OS)	-0,01	0,99	0,98	1,00	-0,99%	-2,15%	0,17%
**Vert**	(Intercept)	1,21	3,35	2,66	4,22	NA		
	Imaging (GAF)	-0,02	0,98	0,97	0,99	**-2,19%**	**-3,41%**	**-0,95%**
	Age	0,00	1,00	1,00	1,01	0,24%	-0,29%	0,77%
	Sex (M)	0,12	1,12	0,94	1,35	12,40%	-6,10%	34,50%
	Phenotype (CORD)	-0,19	0,83	0,68	1,01	-17,10%	-31,80%	0,95%
	Eye (OS)	0,01	1,01	1,00	1,03	1,40%	-0,02%	2,76%

CI: confidence interval
